# Food Alliance’s Mobile Food Community Kitchen and Pop-Up Pantry Model

**DOI:** 10.3390/ijerph23050550

**Published:** 2026-04-24

**Authors:** Margaret Henning, Magdalynn Graul, Kate McAvoy

**Affiliations:** 1 Department of Health Science, Keene State College, Keene, NH 03431, USA; kate.mcavoy@keene.edu; 2 Cheshire Medical Center, Keene, NH 03431, USA; mgraul@cheshire-med.com

**Keywords:** mobile food pantry, rural food insecurity, and barriers to access

## Abstract

**Highlights:**

**Public health relevance—How does this work relate to a public health issue?**

- Addresses Social Determinants of Health (SDOH): This work directly addresses food insecurity—a fundamental social determinant—which currently affects 10% of New Hampshire residents and over 13% of children, impacting long-term health outcomes.
- Targets Rural Health Disparities: The research focuses on the intersection of geographic isolation and resource access, specifically looking at how transportation barriers in rural towns exacerbate nutritional deficiencies.

**Public health significance—Why is this work of significance to public health?**

- Evidence-Based Intervention Validation: It provides empirical evidence that mobile delivery effectively mitigates financial stressors for vulnerable populations.
- Prevention of Chronic Disease: Improving access to consistent nutrition through the Healthy Monadnock Alliance collaboration serves as a primary prevention strategy, potentially reducing the incidence of diet-related chronic conditions among aging adults and low-income families.

**Public health implications—What are the key implications or messages for practitioners, policy makers and/or researchers in public health?**

- Scalability of the Mobile Model: For practitioners and policymakers, the study suggests that “bringing the resource to the people” is a replicable and necessary strategy in rural areas where traditional stationary food pantries may be inaccessible due to a lack of public transit.
- Emphasis on Multi-Sector Collaboration: The research highlights that the success of public health initiatives depends on integrated partnerships among nonprofits (The Community Kitchen), healthcare providers (Cheshire Medical Center), and community alliances to ensure long-term program sustainability.

**Abstract:**

This research, funded by the National Science Foundation (Award #2412054) as part of the NH-LIFT project, provides a critical analysis of a successful public health initiative addressing food insecurity in New Hampshire, which affects nearly 10% of residents and 13.4% of children. The study’s primary objective was to analyze the effectiveness, unique characteristics, and replicability of The Community Kitchen’s Mobile Food Pantry program in collaboration with the Healthy Monadnock Alliance and Cheshire Medical Center. Methods: A survey design was employed over a four-week period (July–August 2025) to collect qualitative data from *n* = 97 voluntary participants attending mobile pantry events in four rural southwest New Hampshire towns, Gilsum, Richmond, Winchester, and Fitzwilliam, during the period of May-June of 2025. The anonymous, 25-question instrument gathered information on program benefits and needed improvements. Results indicate the model is highly effective in mitigating increased financial stressors and overcoming transportation barriers, which are critical challenges for families and aging adults in this rural region. While demonstrating success in promoting local health and well-being, the research also highlights factors crucial for long-term sustainability. This study contributes to an evidence-based public health model suitable for replication in other food-insecure rural communities.

## 1. Introduction

Hunger, sometimes used interchangeably with food insecurity, is a real and enduring problem in the Granite State. This research was conducted as part of NH-LIFT, a project funded by the National Science Foundation (Award #2412054), to analyze a public health initiative, The Community Kitchen’s Mobile Food Pantry program, and its innovative partnership with the Healthy Monadnock Alliance and Cheshire Medical Center. Consistent access to sufficient food for an active and healthy life is known as food security. According to the U.S. Department of Agriculture, Economic Research Service [1], “Food security is defined as a situation that exists when all people, at all times, have physical, social, and economic access to sufficient, safe, and nutritious food that meets their dietary needs and food preferences for an active and healthy life. Four dimensions of food security have been identified at different levels. (1) Availability—National. (2) Accessibility—Household. (3) Utilization—Individual. (4) Stability—may be considered as a time dimension that affects all the levels. All four of these dimensions must be intact for full food security. More recent developments emphasize the importance of sustainability, which may be considered as the long-term time (fifth) dimension to food security” (U.S. Department of Agriculture, Economic Research Service [2]. Food security in the U.S.: Measurement).

Food security is directly associated with health outcomes. The U.S. Department of Agriculture, Economic Research Service stated that “a lack of consistent access to enough food for every person in a household to live an active, healthy life” constitutes food insecurity [2]. Current research highlights that food insecurity functions as a primary social determinant of health, creating a “double burden” for families. Food-insecure adults are nearly two to three times more likely to have diabetes and are at a much higher risk for hypertension and cardiovascular disease. Consistent access also includes food preferences, shifting food security away from simple access to calories and nutrition and toward access to food that is also socially and culturally acceptable. Within the context of diabetes, access to sufficient quantities of food does not necessarily guarantee food security if food quality and preferences are ignored [3].

New Hampshire’s experience with food insecurity closely mirrors national trends driven by federal policy changes. From 2019 to 2021, the state saw its lowest food insecurity rate in 20 years, affecting approximately 30,000 households. This reduction was likely a direct result of temporary COVID-19 relief funds designed to mitigate the pandemic’s economic effects. However, once that temporary aid expired, food insecurity increased; in the 2021–2023 period, the number of food-insecure households rose to an estimated 42,300, effectively returning the state to its pre-pandemic struggle. The report presents its main findings as both a percentage (prevalence rate) and an estimated number of households: according to the National Household Food Insecurity Survey in 2023, 13.5 percent of U.S. households (an estimated 18.0 million households) were food insecure. Additionally, in 2023, 5.1 percent of U.S. households (an estimated 6.8 million households) had very low food security [4].

As of May 2024, New Hampshire has faced a deepening food insecurity crisis, with an estimated 135,200 residents, including 33,720 children, experiencing limited or uncertain access to adequate food—a sharp 43.9% increase from the previous year. This challenge also impacts over 80,251 older adults and contributes to 425,000 residents reporting food insufficiency overall, according to the Gerontology Institute at [5,6]. Based on the Mobile Food Pantry program’s three-year evaluation report “Observational impacts and planning for sustainability beyond 2024”. The cost of living for older adults in Cheshire County is above the national average, which also contributes to this concern. The crisis is driven by factors like poverty, transportation barriers, and inflation, particularly in rural areas where food costs have escalated significantly [6,7]. Nutrition access and security: Adequate nutrition is vital for development, and food insecurity has a devastating and disproportionate effect on children’s health. Children in food-insecure households are more likely than their peers to face developmental delays, display poor academic performance, and develop adverse health outcomes that can negatively affect them into adulthood. Furthermore, food insecurity is associated with higher rates of chronic health conditions and poorer mental health in adults [8,9]. Limited access to both supermarkets and reliable transportation increases the risk of food insecurity. This is a critical challenge in rural New Hampshire, as distance affects residents’ ability to buy fresh food—especially those without personal vehicles.

Using 2019 data, the federal Food Access Research Atlas pinpointed low-income areas in Carroll, Cheshire, Coos, and Grafton counties in which most households were more than 10 miles from a grocery store [1,10,11]. Mapping the food landscape in New Hampshire [12]: The Center for Population Health at Cheshire Medical Center found that 19% of people in the Monadnock region of New Hampshire struggled to afford healthy foods such as fresh fruits and vegetables, a significant increase from 7% in 2020. The main barrier identified was the high cost of healthy foods, reported by 82% of respondents, while 12% cited a lack of transportation as their primary concern [5].

To address food access issues across New Hampshire, particularly in the Monadnock Region, the Monadnock Food Access Alliance, a workgroup of the Healthy Monadnock Alliance with support from Cheshire Medical Center, works to ensure that all community members, regardless of income, can access fresh, locally grown produce. Prior research suggests that mobile food pantries offer an additional resource and a unique approach to addressing food insecurity; however, little is known regarding community members’ use of this food distribution option. There is research to support that these “pantries on wheels” play a fundamental role in feeding hungry people. Research conducted by [13,14,15] supports that mobile pantries can serve the most food-insecure populations and can effectively expand food distribution to highly vulnerable populations, such as seniors and people of color [15].

By partnering with local farms and food suppliers, the Monadnock Food Access Alliance has piloted a Mobile Food Pantry program to increase access to healthy, fresh food options across the Monadnock region. The pilot program was developed by the Alliance and directed by The Community Kitchen of Keene, a local nonprofit food supplier that offers hot meals and pantry staples to individuals and families in need. The program identified volunteers at several key rural sites across the region without an existing local food pantry, and provided pop-up pantry events offering fresh products, including meat, dairy, fruits, and vegetables, to attendees at no cost during the summer and fall months from The Community Kitchen’s supplies. In addition to food resources, the events also offer community connections to other resources, including the Supplemental Nutrition Assistance Program (SNAP), a U.S. federal assistance program designed to support the healthcare and nutrition of low-income pregnant women, breastfeeding women, and children under the age of five, the Program for Women, Infants, and Children (WIC), Housing, Double Up Veggie Bucks, Heating Assistance, Healthy Starts (HCS) and the Pet Food Pantry Program provided by the Monadnock Humane Society.

The Mobile Food Pantry’s initial pilot program has grown significantly since it began in 2021, and in its 2025 season provided over 10,300 estimated meal equivalents to 633 unique individuals comprising 243 unique households across the region. While the Mobile Food Pantry Program cannot meet all food needs for the growing population of food-insecure residents, it strives to make a meaningful, supportive impact in the community. The research presented here focuses on analyzing the program’s effectiveness and its potential for replication at additional sites across the region. The Community Kitchen’s Mobile Food Pantry and Pop-up Food Pantry model represents a valuable, community-centric, and transportation-friendly solution to addressing food access issues in rural New Hampshire. This model demonstrates unique strengths in fostering local engagement and adapting to the specific needs of the communities it serves.

## 2. Materials and Methods

To evaluate the efficacy of local food assistance and understand the unique barriers to food access in rural New Hampshire, a mixed-methods research design was employed. This work was done through 3 lenses: first, a review of prior data on service provision, including the number of community members served over time (2021–2024) was undertaken. Second, qualitative survey data on participants’ experiences were collected, and third, participants’ data were also collected through focus group conversations in 2024. Participants were recruited from individuals attending the mobile food pantries in select towns in southwest New Hampshire, including Gilsum, Richmond, Winchester, and Fitzwilliam. Informed consent was obtained from all survey and dinner participants prior to data collection. To acknowledge the value of lived expertise and the time commitment required for the qualitative sessions, each participating household at the Listening Dinners received a $150 Visa gift card as a stipend.

Qualitative survey: The survey instrument was an evaluation tool comprising 25 questions that covered age, residency, household size, how the Mobile Food Pantry best meets their needs, and what improvements are needed. The survey included multiple-choice questions, Likert-scale items, and open-ended responses. Data was collected over a four-week period using paper surveys. The instrument was adapted from a pre-existing questionnaire (U.S. Department of Agriculture, Economic Research Service, 2026) [16]. Additional questions were developed by the Community Health team and a group of community stakeholders (members of the Food Access Alliance and The Community Kitchen). The focus was to evaluate different aspects of the program and service provision, to provide insight into resource allocation, and to understand community needs.

Focus Group: Ongoing program evaluation and collaborative community design came from a series of focus groups. The focus groups were hosted as “Community Listening Dinners.” Three events were held before the Monadnock Food Pantry (MFP) season began. The events aimed to gather insights from community members who have experienced food insecurity and to triangulate the survey data. The goal was to create a space for individuals to share their experiences and coping strategies related to food insecurity, discuss the perceived role of community assistance programs, and propose ideas for improving food access from the perspective of those most affected.

## 3. Results

The results integrated quantitative data from mobile pantry operations with qualitative insights gathered through community engagement. Participation was entirely voluntary. All survey responses were kept anonymous to encourage candid feedback and mitigate social stigma.

### 3.1. Community Listening Dinners

The Community Listening Dinners were designed to gather insights from community members who have experienced food insecurity. These events provided a dedicated space for individuals to share personal experiences, discuss coping strategies, and evaluate the role of community assistance programs. Participants also proposed grassroots ideas for improving food access from the perspective of those most affected. Participant demographics and methodology: Twenty-five participants attended the events, which were held in local community spaces during a shared meal. Discussions focused on three primary populations of interest: families with children, aging adults, and rural residents facing transportation barriers.

Key themes that emerged included the importance of pairing food services with additional resources, the pervasive lack of transportation, the reliance on informal support systems, and the urgent need for service models that recognize and mitigate the stigma associated with food assistance.

### 3.2. Mobile Pantry Impact

Data from 2025 indicates that 12 mobile pantry events served 589 unique households. In total, 12,633 meal equivalents were distributed to 1407 individuals, including 314 children under the age of 18 and 605 adults aged 60 and over.

The Community Kitchen’s Mobile Food Pantry program effectively reached these vulnerable groups; during the 2024–2025 period, approximately one-third of participants were under the age of 18, and another third were over the age of 65. By bringing food directly to these residents, the model successfully addresses transportation barriers and mitigates financial stressors. However, as the program expands, long-term sustainability must be addressed to ensure continued service [5].

### 3.3. Survey Results: Rural Access and Economic Strain

A total of 97 responses (*n* = 97) were collected over a four-week period in July and August 2025. The results underscore that the rural environment presents distinct food access challenges, often creating conditions consistent with “food deserts.” Survey data suggests that 49% of participants must drive more than 15 min to access food. In the absence of public transit, this geographic barrier necessitates localized and mobile distribution solutions.

Food insecurity in this region is rooted in a complex intersection of demographic vulnerability and acute financial strain. Additionally, a central finding concerns the region’s aging demographic. As residents move into retirement, fixed incomes offer little protection against rising living costs. As the nation’s second-oldest state, New Hampshire faces a growing crisis: 25.5% of its seniors live solo, primarily in rural areas with minimal transportation. Record-high food insecurity is forcing these vulnerable residents to choose between heating their homes and buying groceries [17].

## 4. Discussion

There are key thematic considerations triangulated from qualitative and quantitative data: specifically, the distribution matters as much as the food itself. The local presence and the use of local volunteers in familiar community spaces significantly alleviate the fear of judgment. Results indicate a high demand for “open” environments where seeking help feels like a community interaction rather than a bureaucratic process.

The Mobile Food Pantry events were seen as a valuable resource in the community, particularly as a supplemental resource for households with incomes slightly above the Supplemental Nutrition Assistance Program (SNAP) eligibility threshold. To qualify for benefits, participants must meet certain eligibility standards based on their income, assets, household size, immigration status, and proof of employment. While these households struggle with food insecurity, they often fail to meet the strict criteria regarding assets, income, and employment status required for federal aid. The program successfully filled this void by providing a mix of fresh produce and pantry staples. Many individuals experience nervousness or shame when accessing support. Having local volunteers present in community spaces can help all alleviate these feelings, making it easier for people to reach out without fear of judgment. Participants consistently reported that one-time events are insufficient for long-term stability

Pairing food services with other resources: Connections to other community providers and resources at the mobile pantry events were highly valued. For example, the Humane Society is partnering to provide food in addition to dog beds and food in addition to other resources. Research by [18] indicates that people requested healthy foods but also experienced inconsistent availability and placed the highest value on their autonomy to choose items. Continuous monitoring of these client priorities is therefore essential.

Lack of public transit or people being homebound was a major barrier: food security is closely linked to other life situations, such as taking on debt to get medical care or an unexpected expense like a car repair. There was strong support for the idea of home deliveries or volunteers doing additional food drops to ensure that those who are homebound or without transportation have access to food.

Rural communities often have informal support systems. In this case, participants at the community dinners reported that neighbors share resources, such as food, rides, and other necessities, and coordinate efforts to help each other in times of need. These support systems create a sense of community and resilience, allowing individuals to make the most of what they have. In many communities, informal networks of information and resource sharing play a crucial role. If these roles and systems are understood, there is an opportunity to leverage and better support individuals and families working within existing systems.

## 5. Conclusions

Food insecurity is recognized as a critical population health issue driven by a complex interplay of factors, extending far beyond immediate access to food. Findings from surveys and the “Community Listening Dinners” underscore the critical importance of a service design that is respectful, resource-paired, and stigma-reducing. Participants highly valued the ability to access fresh, locally sourced food alongside essential resource information (SNAP, WIC, etc.). The research suggests that while the MFP cannot fully meet the growing need, its strength lies in fostering local engagement and acting as a supplemental resource for households facing financial strains and logistical hurdles.

Ultimately, the MFP model offers a strong, evidence-based approach to promoting public health in rural New Hampshire, particularly by overcoming transportation limitations and fostering robust, informal community support systems. Ensuring its long-term sustainability is key, as this innovative, collaborative program offers a replicable model for other rural regions struggling with the complex intersection of food access, chronic health conditions, and demographic vulnerability.

## Data Availability

The original contributions presented in this study are included in the article. Further inquiries can be directed to the corresponding author.
